# Whole Genome Analysis and Prognostic Model Construction Based on Alternative Splicing Events in Endometrial Cancer

**DOI:** 10.1155/2019/2686875

**Published:** 2019-07-02

**Authors:** Caixia Wang, Mingjun Zheng, Shuang Wang, Xin Nie, Qian Guo, Lingling Gao, Xiao Li, Yue Qi, Juanjuan Liu, Bei Lin

**Affiliations:** ^1^Department of Obstetrics and Gynaecology, Shengjing Hospital Affiliated to China Medical University, Liaoning, China; ^2^Key Laboratory of Maternal-Fetal Medicine of Liaoning Province, Key Laboratory of Obstetrics and Gynecology of Higher Education of Liaoning Province, Liaoning, China

## Abstract

**Objectives:**

A growing body of evidence has shown that aberrant alternative splicing (AS) is closely related to the occurrence and development of cancer. However, prior studies mainly have concentrated on a few genes that exhibit aberrant AS. This study aimed to determine AS events through whole genome analysis and construct a prognostic model of endometrial cancer (EC).

**Methods:**

We downloaded gene expression RNAseq data from UCSC Xena, and seven types of AS events from TCGA SpliceSeq. Univariate Cox regression was employed to analyze the prognostic-related alternative splicing events (PASEs) and splicing factors; multivariate Cox regression was conducted to analyze the effect of risk score (All) and clinicopathological parameters on EC prognosis. An underlying interaction network of PASEs of EC was constructed by Cytoscape Reactome FI, GO, and KEGG pathway enrichment was performed by DAVID. ROC curves and Kaplan-Meir analysis were used to assess the diagnostic value of prognostic model. The correlation between PASEs and splicing factors was analyzed by GraphPad Prism; then a network was constructed using Cytoscape.

**Results:**

In total, 28,281 AS events in EC were identified, which consisted of 1166 PASEs. RNPS1, NEK2, and CTNNB1 were the hub genes in the network of the top 600 PASEs. The area under the curve (AUC) of risk score (All) reached 0.819. Risk score (All) together with FIGO stage, cancer status, and primary therapy outcome success was risk factors of the prognosis of EC patients. Splicing factors YBX1, HNRNPDL, and HNRNPA1 were significantly related to the overall survival (OS). The splicing network indicated that the expression of splicing factors was significantly correlated with percent-splice-in (PSI) value of PASEs.

**Conclusion:**

We constructed a model for predicting the prognosis of EC patients based on PASEs using whole genome analysis of AS events and thereby provided a reliable theoretical basis for EC clinical prognosis evaluation.

## 1. Introduction

Alternative splicing (AS) is an important regulation mechanism in the process of mRNAs after transcription. Pre-mRNA produces different mRNAs through different splicing methods to translate into different proteins with versatile functions, which contributes to protein diversity [1]. AS is a ubiquitous biological process, approximately 95% of human genes undergo AS in physiological processes [2]. In recent years, studies have shown that AS plays an important role in the occurrence and development of cancer, and AS participates in the process of proliferation, apoptosis, and metastasis of tumor cells [3, 4]. AS is mainly regulated by the spliceosome, which is the complex proteins composed of small nuclear ribonucleic acids (snRNA) and splicing factors [5]. A splicing factor is an important accessory protein that regulates pre-mRNA AS. Mutation and/or abnormal expression of splicing factors are closely related to the occurrence of abnormal AS [6]. In total, there are seven types of AS events listed in the SpliceSeq database, i.e., alternate acceptor site (AA), alternate donor site (AD), alternate terminator (AT), alternate promoter (AP), exon skip (ES), mutually exclusive exons (ME), and retained intron (RI) [7].

Endometrial cancer (EC) is one of the common gynecological malignant tumors. In recent years, older age (*⩾*55 years old), obesity (*⩾*25 kg/m^2^), diabetes, and tamoxifen adjuvant therapy have led to an increase in the incidence of endometrial cancer (EC) [8]. According to cancer statistics in 2019, an estimated 61,880 new cases of EC will occur in the United States, proceeding to the first in gynecological malignant tumors [9]. EC is a hormone-dependent cancer: research has shown that AS of estrogen receptor *α* (ER*α*) and progesterone receptor (PR) is closely related to the occurrence and development of EC [10]. A newly defined splicing factor, YT521, was found to promote the AS of vascular endothelial growth factor A (VEGF-A), thereby leading to an increase in splicing variant VEGF-165 and ultimately promoting EC invasion [11]. Until now, monitoring the diagnosis and prognosis of EC has been limited by a lack of biomarkers with both sensitivity and specificity. Therefore, the purpose of our study was to elucidate aberrant AS in EC, which would provide a theoretical basis for finding prognostic biomarkers and therapeutic targets for EC. We also analyzed PASEs through whole genome analysis and constructed prognostic model for EC.

In the present study, we obtained normalized RNA-seq data from UCSC Xena and seven types of AS events from TCGA SpliceSeq database, then performed a systematic analysis to figure out PASEs in EC, then we constructed prognostic model risk score (All) based on PASEs, and evaluated the diagnostic value of risk score (All) in assessing the prognosis of EC. In addition, splicing factors that were associated with prognosis of EC were identified using univariate Cox regression and a correlation network between splicing factors and PASEs was constructed. In summary, our study developed a new prognostic model for EC.

## 2. Materials and Methods

### 2.1. Data Extraction and Preprocessing

RNAseq-HTSeq-FPKM-UQ data was downloaded from UCSC Xena (https://xenabrowser.net/), which provided normalized RNAseq data. Except for 35 cases of adjacent normal samples, seven types of AS events of 545 ECs were downloaded from the TCGA SpliceSeq (https://bioinformatics.mdanderson.org/TCGASpliceSeq/index.jsp) database, and PSI value ranges from 0 to 1, which is used to quantify AS events. AS events with PSI range ≥ 0.5 were included in this study.

### 2.2. Building a Prognostic Model Based on PASEs

Patients with overall survival (OS) ≥ 30 days were included in this study. According to its median number, each parameter was divided into high-risk (≥ median number) and low-risk (<median number) groups. Univariate Cox regression was performed on all seven types of AS events, and lnHR was used as the *β*-coefficient to construct a prognostic model for EC [12]. The prognostic model was constructed according to

(1) $$\begin{array}{c} Risk\;\;score=\overset{n}{\underset{i}{\sum }}PSIi*\beta i \end{array}$$

### 2.3. Evaluation of the Diagnostic Value of Prognostic Models

A Kaplan-Meier survival curve was plotted using the “survival” package (version 2.43-3) to assess the effect of the prognostic model on the 5-year OS of EC. A ROC curve was drawn using the “survivalROC” package (version 1.0.3) to assess the diagnostic value of prognostic models of EC

### 2.4. Functional and Pathway Enrichment Analysis

DAVID (https://david.ncifcrf.gov/) is a database that integrates biological data and analysis tools and provides comprehensive annotation information. PASEs were analyzed using DAVID (version 6.8), including GO terms (Biological Process/Cellular Component/Molecular Function, BP/CC/MF) and KEGG pathways.

### 2.5. Construction of an Interaction and Correlation Network

To further explore the interaction of PASEs in EC, we constructed an interaction network using Cytoscape Reactome FI (version 3.6.0), and hub genes were selected according to the number of degrees. The splicing factors were downloaded from the TCGA SplieAid2 (http://193.206.120.249/splicing_tissue.html) database (Table S1), and a scatter plot between prognostic-related splicing factor expression profiles and the PSI values of the selected PASEs was constructed in GraphPad Prism8.0. The correlation network between splicing factors and PASEs was constructed in Cytoscape (version 3.7.1).

### 2.6. Statistical Analysis

UpSetR (version 1.3.3) was used to quantify seven types of PASEs in EC. Statistical analyses were performed using R/Bioconductor (version 3.4.3) and GraphPad Prism (version 8.0). Two-tailed* P *< 0.05 was considered statistically significant. ∗* P *< 0.05, ∗∗* P *< 0.01, and ∗∗∗* P *< 0.001.

## 3. Results

### 3.1. Overview of AS Events in TCGA-EC

**Figure 1:**
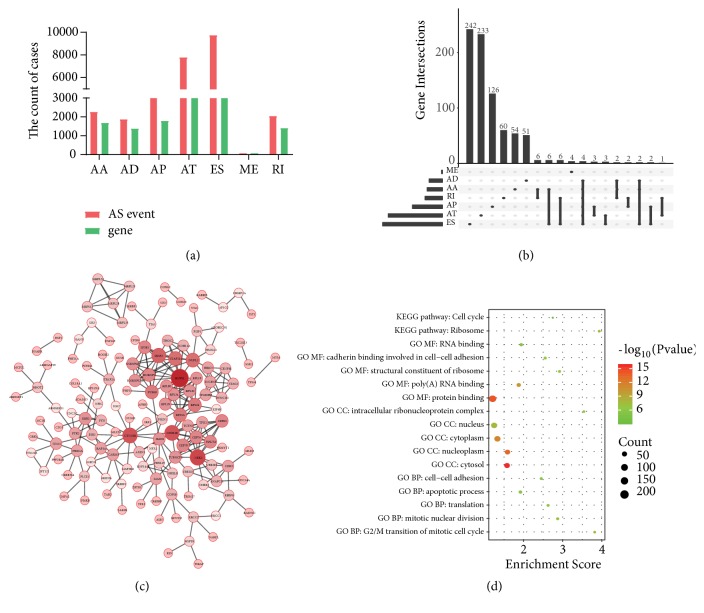
Upset and bioinformatic analysis of PASEs in EC. (a) AS events and genes in seven types of alternative splicing. (b) Upset plot of PASEs in EC. (c) Interaction network of PASEs constructed by cytoscape Reactome FI. (d) The bubble plot of Top5 GO (BP/CC/MF) and KEGG pathway.

In total, there were 8140 genes, and 28281 AS events in 545 EC patients and the median number of AS events per gene was 3.474. Among the seven types of AS events, ES was the most common, followed by AT and AP. Specifically, there were 4604 genes in the 9744 ES events, 3411 genes in the 7796 ATs event, 1792 genes in the 4458 AP events, 1691 genes in the 2270 AA events, 1413 genes in the 2050 RI events, 1386 genes in the 1877 AD events, and 85 genes in the 86 ME events (Figure 1(a)).

It is worth noting that one gene can undergo multiple types AS events; therefore, UpSet picture was used to match the genes with AS events. The results showed that the most common event was ES followed by AT and AP; ME events occurred the least. Three (AA, AD, and ES) AS events observed were DMKN and ATXN2L (Figure 1(b)).

To better track the AS events, we named AS events using the gene name, AS type, and a unique ID number in AS event. For example, in RPLP0-ES-24731, RPLP0 represents the gene name, ES represents the type of AS event, and 24731 was the unique ID number of the AS event.

### 3.2. AS Events Associated with Prognosis of EC

To build a prognostic model, we needed to find AS events with good discrimination; thus, AS events with PSI range ≥ 0.5 were included in this study. Ultimately, we obtained 5015 genes and 11709 AS events.

To explore the relationship between AS events and EC prognosis, we evaluated prognostic AS events using univariate Cox regression. The results showed that 1166 AS events were significantly associated with OS in EC (*P*<0.05; Table S2). We selected top 600 PASEs and constructed an interaction network using Cytoscape Reactome FI. The results showed that the RNPS1, NEK2, and CTNNB1 genes were the hub nodes of this network (Figure 1(c)). Then we performed GO (BP/CC/MF) and KEGG pathway analysis using the DAVID database and found that 19 BPs were enriched, such as the G2/M transition of mitotic cell cycle, mitotic nuclear division, and translation, 12 CCs, 12 MFs (*P*<0.05), and 2 KEGG signaling pathways (*P*<0.05; Table S3), such as ribosome and cell cycle, and plotted the bubbles of Top5 GO (BP/CC/MF) and the KEGG pathways (Figure 1(d)).

### 3.3. Construction of Prognostic Model of EC

To investigate the relationship between AS events and EC survival outcomes, we constructed a prognostic model of EC based on PASEs. The* P *values were sorted from small to large, and the top 4 genes for each AS event were selected to construct a prognostic model, while the top 6 genes were used to construct the risk score (All) prognostic model. We chose lnHR to construct a formula of prognostic models (Table 1).

Risk score (All) = (PSI value of RPLP0-ES-24731 × -9.093) + (PSI value of ZNF586-AT-52338 × 2.913) + (PSI value of STK32C-AP-13486 × -2.391) + (PSI value of C4orf29-AT-70557 × 4.133 ) + (PSI value of C4orf29-AT-70558× -4.133 ) + (PSI value of ANAPC11-ES-44217 × 3.945).

**Figure 2:**
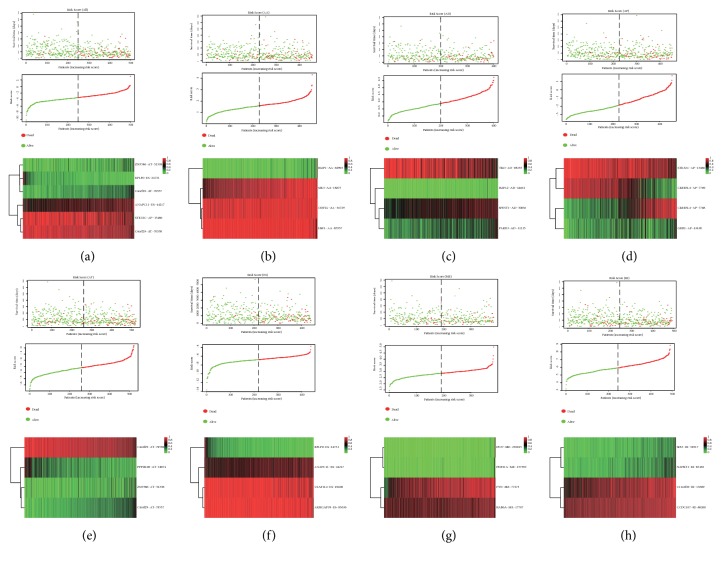
Construction of eight PASEs models for EC. (a–h) Risk scores for All, AA, AD, AP, AT, ES, ME, RI models in EC, respectively. Each individual plot (Top) represents the distribution of survival time and survival status of high- and low-risk groups. (middle) represents the distribution of patients in the high- and low-risk groups, (bottom) represents the PSI value heat map of the alternative splicing genes in the constructed model.

**Figure 3:**
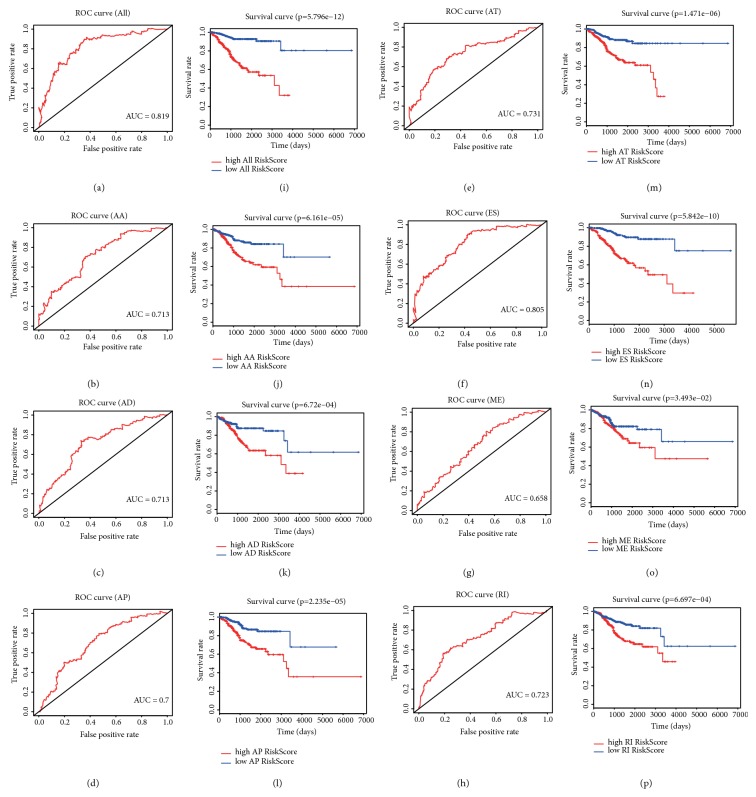
ROC curves and K-M survival curves for eight PASEs models in EC. (a–h) ROC curves for All, AA, AD, AP, AT, ES, ME, RI models. (i–p) K-M survival curves of All, AA, AD, AP, AT, ES, ME, RI models.

According to the median number of risk score, EC patients were divided into high and low-risk groups, which indicated that the mortality rate was higher for EC patients in the high-risk group (red spots), which was associated with the lower OS (red and green spots; Figures 2(a)–2(h)). A ROC curve was used to evaluate the diagnostic value of the prognostic model; results suggest that the AUC of the risk score (All) is 0.819 followed by 0.805 for the risk score (ES) and 0.731 for the risk score (AT) (Figures 3(a)–3(h)). Compared with the prognostic model constructed by a single AS event, risk score (All) better predicted EC patients' prognosis. We also plotted survival curves for all prognostic models (Figures 3(i)–3(p)) and found that the high-risk groups of all prognostic models were associated with poor prognosis in EC patients (*P*<0.05).

In addition, we analyzed the relationship between clinicopathological parameters and EC prognosis. Age more than 55 years old and BMI more than 25 kg/m^2^ were considered as high-risk factors. Univariate Cox regression analysis indicated that pathological type, FIGO stage, grade, cancer status, new tumor event after initial treatment, primary therapy outcome success, and risk score (All) were associated with poor prognosis of EC patients (*P*<0.05). Multivariate Cox regression showed that only FIGO stage, cancer status, primary therapy outcome success, and risk score (All) were risk factors for the poor prognosis of EC (*P*<0.05) (Table 2).

### 3.4. Splicing Factors Associated with EC Prognosis

**Figure 4:**
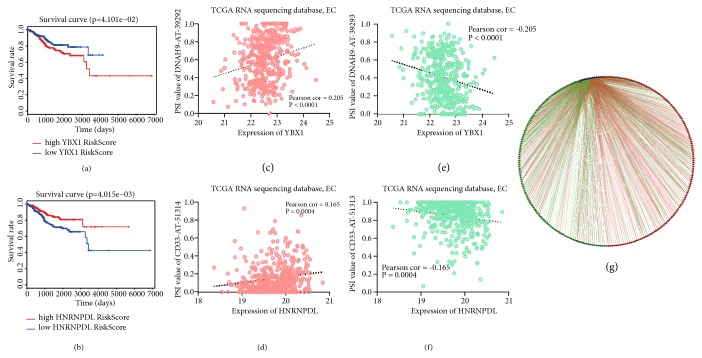
Correlation between prognosis-related splicing factors and PASEs in EC. (a, b) K-M survival curves of YBX1 and HNRNPDL, divided into high (red line), low (green line) group according to median expression value of splicing factor. (c, e) Correlation analysis between expression of YBX1 and PSI values of DNAH9-AT-39292, DNAH9-AT-39293. (d, f) Correlation analysis between expression of HNRNPDL and PSI values of CD33-AT-51314, CD33-AT-51313. (g) Correlation network between prognosis-related splicing factors and PSI values of alternative splicing events, blue dots represent splicing factors, green/red dots represent favorable/poor prognostic alternative splicing events, respectively, and red/green lines represent positive/negative correlations, respectively.

Splicing factors are the executors of the AS events, and mutation or abnormal expression of splicing factors are related to the occurrence and development of cancer. To identify prognosis-associated splicing factors in EC, we performed univariate Cox regression analysis and obtained 10 splicing factors (FMR1, SRSF4, PCBP2, PTBP2, HNRNPDL, YBX1, QKI, RBM5, HNRNPA1, and HNRNPF;* P*<0.05), among which splicing factors SRSF4, HNRNPDL, RBM5, HNRNPA1, and HNRNPF were considered protective factors (Table S4). A correlation network constructed by Cytoscape indicated that 10 splicing factors (blue dots) were negatively (green lines) correlated with 125 favorable prognostic AS events (green dots), and positively correlated (red lines) with 143 AS events with poor prognosis (red dots; Figure 4(g); Table S5).

We constructed Kaplan-Meier survival curves of 10 splicing factors and found that only YBX1, HNRNPDL, and HNRNPA1 had significant effects on OS in EC patients (Figure S1). We plotted representative K-M survival curves of YBX1 and HNRNPDL (*P*<0.05; Figures 4(a) and 4(b)). In addition, we generated a scatter plot between the expression of splicing factors and PSI value of AS events and found that splicing factor YBX1 was positively correlated with PSI value of DNAH9-AT-39292 and negatively correlated with PSI value of DNAH9-AT-39293 (Figures 4(c) and 4(e)). Splicing factor HNRNPDL showed different correlation between different splicing event types of the same gene CD33 (*P*<0.005; Figures 4(d) and 4(f)). The above findings suggested that different splicing factors played different roles in different AS events.

## 4. Discussion

AS is an important biological process for producing protein diversity. Abnormal AS events in tumors are closely related to tumor initiation and tumor progression. A gene can undergo different types of AS events and can be regulated by a variety of splicing factors, thus complicating the study of the regulatory networks between AS events and splicing factors. At present, research mainly detected AS of EC by using small sample sequencing [13]. However, studies employing whole genome analysis of AS events in EC have not been reported. In recent years, high-throughput sequencing technology can better characterize abnormally mutated splices and splice sites; therefore, it is important to use bioinformatics techniques to analyze abnormal AS events in EC.

In the present study, we downloaded seven types of AS events from the TCGA SpliceSeq database. There were 8140 genes and 28281 AS events in EC, which indicates that AS events are ubiquitous in EC. 1166 PASEs were identified using univariate Cox regression analysis, and the underlying regulatory network was constructed using Cytoscape based on the top 600 PASEs. The results indicated that the RNPS1, NEK2, and CTNNB1 genes were hub nodes of the network. RNPS1 is a member of the SR protein family and functions as an activator in the AS process [14, 15]. RNPS1 can inhibit abnormal splicing of pre-mRNA and plays an important role in quality control during pre-mRNA splicing [15, 16]. Nek2 is a serine/threonine protein kinase that is involved in the regulation of the cell cycle, and there exist three alternative splice variants, Nek2A, Nek2B, and Nek2C [17]. A prior study showed that Nek2C was highly expressed in breast cancer cells and promoted breast cancer cells invasion and migration [18]. In addition, we performed GO (BP/CC/MF) and KEGG pathway enrichment using DAVID and found that these genes were closely related to transition of mitotic cell cycle, mitotic nuclear division, ribosome, cell cycle, etc. From the above perspective, RNPS1, NEK2, and CTNNB1 were the hub genes in the identified network and might therefore be novel targets for EC treatment.

Recently, a prognostic model was constructed based on four miRNAs (miR-4758, miR-876, miR-142, and miR-190b) to evaluate the prognosis of EC patients [19]. However, the construction of a prognostic model for EC based on AS events has not been reported. To assess the diagnostic value of aberrant AS events in the prognosis of EC, we constructed prognostic models based on risk score (All) and seven types of AS event types (AA, AD, AP, AT, ES, ME, and RI). EC patients were divided into high-risk and low-risk groups according to the median number of each AS event, then we plotted Kaplan-Meier survival curves of risk score (All) and the risk scores of each of the seven types of AS events. The results showed that risk score (All) and seven types of AS events were associated with poor prognosis in EC patients (*P*<0.05). In addition, we also plotted ROC curves. The results show that the AUC of the risk score (All) is 0.819 followed by 0.805 for the risk score (ES) and 0.731 for the risk score (AT). Univariate Cox regression was used to analyze the effects of clinicopathological parameters and risk score (All) on the prognosis of EC patients, and multivariate Cox regression was performed to analyze the risk factors that were associated with prognosis. The results show that FIGO stage, cancer status, primary therapy outcome success, and risk score (All) were risk factors for EC patients. These results suggested that the risk score (All) model played an important role in the assessment of EC prognosis.

Abnormalities in splicing factor expression are also related to aberrant AS events. In this study, we obtained 10 splicing factors that are related to the OS of EC patients using univariate Cox regression. By plotting Kaplan-Meier survival curves, we found that only splicing factors YBX1, HNRNPDL, and HNRNPA1 were significantly associated with EC prognosis (*P*<0.05). The constructed splicing network analyzes the association between splicing factors and PASEs in EC. The results suggested that poor prognostic AS events were positively correlated with the expression of splicing factors, while favorable prognostic AS events was negatively correlated with the expression of splicing factors. A splicing factor can precisely regulate the pre-mRNA splicing process by binding to the splicing regulatory sequence elements of a particular gene [20]. Y-box binding protein (YBX1) is a DNA/RNA-binding protein that is involved in gene transcriptional regulation and pre-mRNA splicing [21–23]. Allemand E [24] found that YBX1 interacted with the active form of PP2Cgamma, which is involved in spliceosome assembly, thus regulating CD44 splicing. In breast cancer, it has been shown that there is a significant difference in the expression of YBX1 between triple-negative (TN) and ER+ subtypes [25]. Additionally, another study found that high expression of YBX1 in ER+ breast cancer patients was associated with poor prognosis [26]. It is well-known that EC is a hormone-dependent tumor. EC is mainly divided into estrogen-dependent (type I) and non-estrogen-dependent (type II), and studies have shown that an ER- and PR-type is associated with poor prognosis of EC patients [27]. Therefore, we hypothesized that the differential expression of splicing factor YBX1 was closely related to the different types of endometrial cancer. Interestingly, our study showed that YBX1 was both positively and negatively correlated with different splicing patterns of axonemal dynein heavy chain (DNAH9), which indicated that YBX1 might regulate the splicing of DNAH9. In summary, YBX1 might be diagnostic biomarker and therapeutic target for EC.

HNRNPDL and HNRNPA1 are members of the hnRNP family of RNA-binding proteins that are involved in RNA maturation and translation and in pre-mRNA AS [28, 29]. Studies have shown that the inhibition of HNRNPDL expression can result in increased expression of genes that are involved in cell proliferation and migration [28]. Consistent with this research, our results indicated that HNRNPDL served as a potent tumor suppressor gene. However, studies have shown that HNRNPA1 is highly expressed in gastric cancer cells, thus promoting gastric cancer proliferation, invasion, migration, and EMT [1, 30]. HNRNPA1 regulated AS of the androgen receptor (AR) and gave rise to a splice variant AR-V7, which conferred drug resistance toward enzalutamide in cancer cells [31]. The aforementioned studies collectively indicated that high expression of HNRNPA1 could promote tumor progression. Our results indicated that high expression of the splicing factors HNRNPA1 was a protective factor for the prognosis of EC patients. We hypothesized that HNRNPA1 may have different expression patterns at the tissue level and might exert different biological functions. Further experimental verification of HNRNPA1 should be pursued in EC.

## 5. Conclusions

In summary, we analyzed the effects of abnormal AS events and splicing factors on the prognosis of EC through whole genome analysis and constructed a new clinical prognosis prediction model for EC based on risk score (All). In addition, we built a correlation network between splicing factors and PASEs, which was important to investigate the potential regulatory mechanisms between splicing factors and PASEs. Altogether, our study provided a new theoretical basis for prognostic evaluation and targeted therapy for EC.

## Figures and Tables

**Table 1:** Construction of prognostic model of EC.

Type	ID	*β*	HR	Lower	Upper	*P* value
All	RPLP0-ES-24731	-9.093	1.12E-04	1.15E-06	0.011	1.00E-04 ∗∗∗
	ZNF586-AT-52338	2.913	18.42	4.238	80.06	1.02E-04 ∗∗∗
	STK32C-AP-13486	-2.391	0.092	0.027	0.31	1.21E-04 ∗∗∗
	C4orf29-AT-70557	4.133	62.39	7.408	525.417	1.44E-04 ∗∗∗
	C4orf29-AT-70558	-4.133	0.016	1.90E-03	0.135	1.44E-04 ∗∗∗
	ANAPC11-ES-44217	3.945	51.663	6.701	398.295	1.53E-04 ∗∗∗
AA	ODF2L-AA-3672	2.0766	7.977	2.699	23.575	1.73E-04 ∗∗∗
	HSF1-AA-85557	-3.667	0.026	3.61E-03	0.181	2.39E-04 ∗∗∗
	BMP1-AA-82993	3.809	45.089	5.254	386.93	5.15E-04 ∗∗∗
	SIK3-AA-18875	3.627	37.604	4.795	294.899	5.57E-04 ∗∗∗
AD	IMPA2-AD-44661	4.107	60.745	4.778	772.247	1.55E-03 ∗∗
	SPINT1-AD-30056	2.654	14.215	2.724	74.17	1.64E-03 ∗∗
	TRO-AD-89255	-1.461	0.232	0.093	0.579	1.73E-03 ∗∗
	PARD3-AD-11215	2.814	16.673	2.828	98.309	1.88E-03 ∗∗
AP	STK32C-AP-13486	-2.391	0.092	0.027	0.31	1.21E-04 ∗∗∗
	CREB3L4-AP-7768	1.379	3.972	1.942	8.125	1.58E-04 ∗∗∗
	CREB3L4-AP-7769	-1.379	0.252	0.123	0.515	1.58E-04 ∗∗∗
	GRB2-AP-43438	2.56	12.929	3.392	49.286	1.78E-04 ∗∗∗
AT	ZNF586-AT-52338	2.913	18.42	4.238	80.06	1.02E-04 ∗∗∗
	C4orf29-AT-70557	4.133	62.39	7.408	525.417	1.44E-04 ∗∗∗
	C4orf29-AT-70558	-4.133	0.016	1.90E-03	0.135	1.44E-04 ∗∗∗
	PPP2R1B-AT-18674	-4.078	0.017	2.01E-03	0.143	1.76E-04 ∗∗∗
ES	RPLP0-ES-24731	-9.093	1.12E-04	1.15E-06	0.011	1.00E-04 ∗∗∗
	ANAPC11-ES-44217	3.945	51.663	6.701	398.295	1.53E-04 ∗∗∗
	U2AF1L4-ES-49268	-3.799	0.022	3.08E-03	0.163	1.77E-04 ∗∗∗
	ARHGAP39-ES-85636	-4.678	9.29E-03	7.91E-04	0.109	1.98E-04 ∗∗∗
ME	IPO7-ME-250015	3.358	28.742	4.152	198.957	6.69E-04 ∗∗∗
	FYN-ME-77273	1.682	5.377	1.445	20.006	0.012 ∗
	PHF21A-ME-157593	3.419	30.549	1.735	537.811	0.019 ∗
	RAB6A-ME-17707	2.375	10.75	1.085	106.502	0.042 ∗
RI	SIX5-RI-50517	3.467	32.036	4.378	234.442	6.40E-04 ∗∗∗
	C11orf49-RI-15609	2.418	11.219	2.8	44.957	6.41E-04 ∗∗∗
	CCDC107-RI-86260	4.666	106.224	6.956	1622.054	7.95E-04 ∗∗∗
	NAPRT1-RI-85430	2.911	18.371	2.992	112.814	1.67E-03 ∗∗

**Table 2:** Univariate and multivariate Cox regression analysis of clinicopathological parameters and risk score (All) in 523 EC patients.

Variables	Group	Patient N=523	Univariate analysis		Multivariate analysis	
			HR (95% CI)	*P *value	HR (95% CI)	*P *value
Age		521	1.607(0.83-3.112)	0.16		
	<55	94				
	≥55	427				
BMI		492	0.995 (0.576-1.718)	0.986		
	<25	91				
	≥25	401				
Subtype		523	2.993(1.96-4.57)	3.85E-07 ∗∗∗	1.332(0.626-2.835)	0.456
	Endometrioid	393				
	Non-Endometrioid	130				
Stage		523	4.123(2.691-6.316)	7.55E-11 ∗∗∗	2.832(1.362-5.888)	0.005∗∗
	I-II	378				
	III-IV	145				
Grade		523	11.692(2.876-47.53)	5.90E-04 ∗∗∗	1.32 (0.533-3.274)	0.549
	Low grade (G1)	96				
	High grade (G2/G3)	427				
Cancer status		495	9.018(5.817-13.98)	<2E-16 ∗∗∗	2.282(1.094-4.763)	0.028 ∗
	Tumor free	417				
	With tumor	78				
New tumor event after initial treatment		467	5.735 (3.54-9.292)	1.31E-12 ∗∗∗	1.738(0.9-3.359 )	0.1
	NO	387				
	YES	80				
Primary therapy outcome success		417	9.312(4.936-17.56)	5.54E-12 ∗∗∗	4.15 (1.741- 9.892)	1.33E-03 ∗∗
	CRR+SD+PRR	401				
	PD	16				
Radiation		505	0.64(0.404-1.016)	0.0586		
	NO	278				
	YES	227				
Risk score (All)		499	5.823 (3.308-10.25)	1.02E-09 ∗∗∗	3.548(1.6-8.02)	2.05E-03 ∗∗
	Low	250				
	high	249				

Abbreviations: BMI, Body Mass Index; CRR, Complete Remission/Response; SD, Stable Disease; PRR, Partial Remission/Response; PD, Progressive Disease.

## Data Availability

The data used to support the findings of this study are included within the article.

## Supplementary Materials

Supplementary 1 Figure S1. Kaplan-Meier survival curves of 10 splicing factors in EC. (A-J) K-M survival curves of FMR1, SRSF4, PCBP2, PTBP2, HNRNPDL, YBX1, QKI, RBM5, HNRNPA1, and HNRNPF, respectively.

Supplementary 2 Table S1. Splicing factors and corresponding gene symbols.

Supplementary 3 Table S2. 1166 prognostic-related alternative splicing events with unique TCGA SpliceSeq AS id in EC.

Supplementary 4 Table S3. GO (BP/CC/MF) and KEGG pathway enrichment using the DAVID database.

Supplementary 5 Table S4. 10 prognosis-associated splicing factors in EC.

Supplementary 6 Table S5. Correlation matrix between PASEs and splicing factors.
