# Supplementary figures and images for: Whole Genome Analysis and Prognostic Model Construction Based on Alternative Splicing Events in Endometrial Cancer
Source: Biomed Res Int. 2019 Jul 2;2019:2686875. doi: 10.1155/2019/2686875 (PMC6634061; doi:10.1155/2019/2686875)

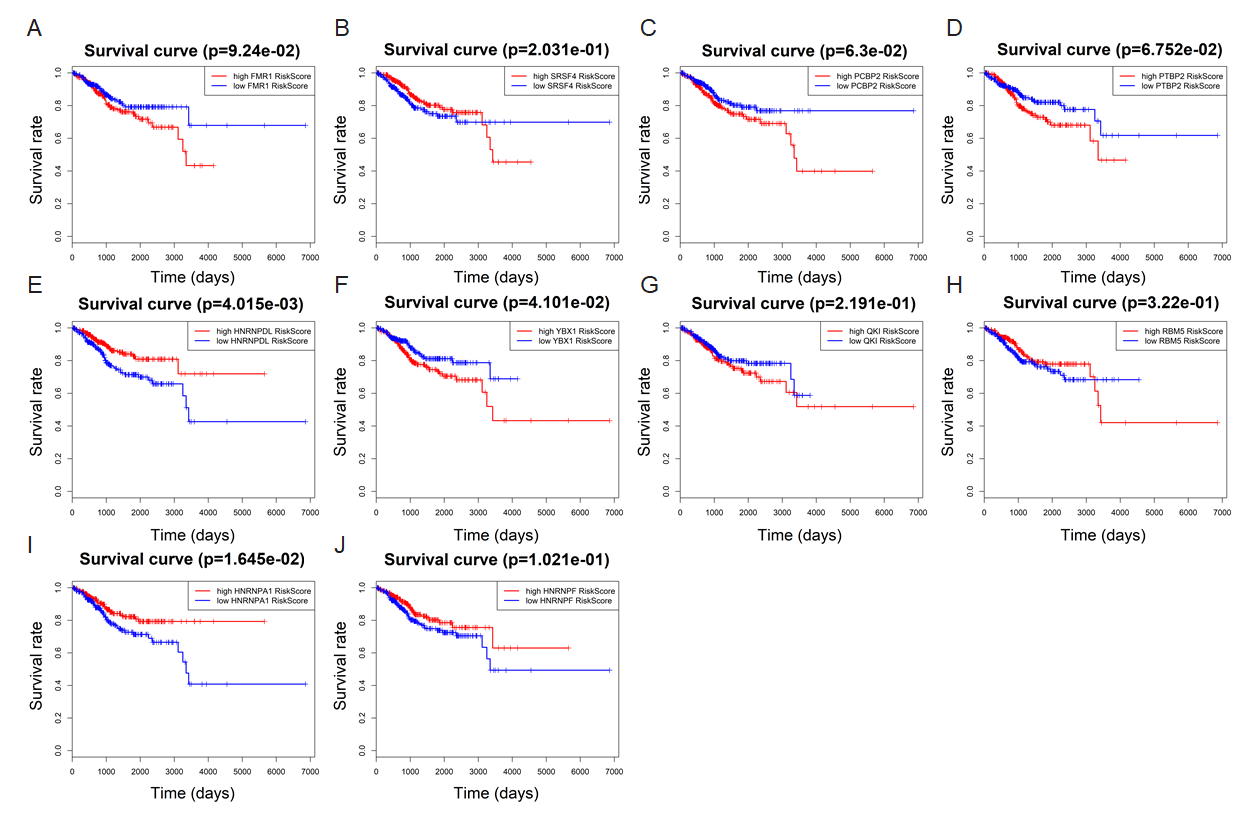

Supplement: Supplementary 1 — Figure S1. Kaplan-Meier survival curves of 10 splicing factors in EC. (A-J) K-M survival curves of FMR1, SRSF4, PCBP2, PTBP2, HNRNPDL, YBX1, QKI, RBM5, HNRNPA1, and HNRNPF, respectively. [file 2686875.f1.tif]
